# Syrian refugees in Greece: experience with violence, mental health status, and access to information during the journey and while in Greece

**DOI:** 10.1186/s12916-018-1028-4

**Published:** 2018-03-13

**Authors:** Jihane Ben Farhat, Karl Blanchet, Pia Juul Bjertrup, Apostolos Veizis, Clément Perrin, Rebecca M. Coulborn, Philippe Mayaud, Sandra Cohuet

**Affiliations:** 1 Epicentre, Paris, France; 2 London School of Hygiene & Tropical Medicine, London, UK; 3 Médecins Sans Frontières Greece, Athens, Greece; 4 Médecins Sans Frontières France, Paris, France

**Keywords:** Refugees, Migrants, Europe, Greece, Syria, Mental health, Violence, Journey, Access

## Abstract

**Background:**

Since 2015, Europe has been facing an unprecedented arrival of refugees and migrants: more than one million people entered via land and sea routes. During their travels, refugees and migrants often face harsh conditions, forced detention, and violence in transit countries. However, there is a lack of epidemiological quantitative evidence on their experiences and the mental health problems they face during their displacement. We aimed to document the types of violence experienced by migrants and refugees during their journey and while settled in Greece, and to measure the prevalence of anxiety disorders and access to legal information and procedures.

**Methods:**

We conducted a cross-sectional population-based quantitative survey combined with an explanatory qualitative study in eight sites (representing the range of settlements) in Greece during winter 2016/17. The survey consisted of a structured questionnaire on experience of violence and an interviewer-administered anxiety disorder screening tool (Refugee Health Screener).

**Results:**

In total, 1293 refugees were included, of whom 728 were Syrians (41.3% females) of median age 18 years (interquartile range 7–30). Depending on the site, between 31% and 77.5% reported having experienced at least one violent event in Syria, 24.8–57.5% during the journey to Greece, and 5–8% in their Greek settlement. Over 75% (up to 92%) of respondents ≥15 years screened positive for anxiety disorder, which warranted referral for mental health evaluation, which was only accepted by 69–82% of participants. Access to legal information and assistance about asylum procedures were considered poor to non-existent for the majority, and the uncertainty of their status exacerbated their anxiety.

**Conclusions:**

This survey, conducted during a mass refugee crisis in a European Community country, provides important data on experiences in different refugee settings and reports the high levels of violence experienced by Syrian refugees during their journeys, the high prevalence of anxiety disorders, and the shortcomings of the international protective response.

## Background

Since 2015, Europe has faced an unprecedented arrival of refugees and migrants. According to the United Nations High Commissioner for Refugees (UNHCR), around 1,015,000 refugees and migrants made the dangerous crossing of the Mediterranean Sea in 2015 [1], 362,753 in 2016, and 55,215 from January 1 to May 16, 2017 [2]. Furthermore, the International Organization for Migration reports that 34,887 refugees and migrants traveled by land from Turkey to Bulgaria and Greece in 2015 [3], 24,338 in 2016 [4], and 1205 in the first quarter of 2017 [5]. Syrians accounted for 50% [6], 46% [7], and 36% [5], in 2015, 2016, and 2017, respectively, of these refugees and migrants. The ongoing Syrian war continues to account for the largest number of new refugees and internal and external displacements worldwide [6].

In early 2016, the European Union (EU) reached an agreement with Turkey aimed at stopping the massive influx of refugees and migrants into the Union. Under the deal, the EU and Turkey agreed that all new migrants crossing from Turkey to the Greek islands after 20 March 2016 would be returned to Turkey if they were not applying or not eligible for asylum, or were asylum seekers whose application was considered inadmissible in the EU [8]. The arrangement also included legal channels of resettlement of certain refugees and migrants to the EU, applicable to Syrian refugees. A start date of 4 April 2016 was set for the repatriation/deportation or resettlement of refugees and migrants. However, as of early 2017, less than 10% of the asylum seekers that the EU had committed to resettle had been relocated [9]. Ultimately, tens of thousands of people are currently stranded in Greece, living in difficult conditions, as they await resettlement, repatriation/deportation, or asylum decisions. Exacerbating the situation, Greece has faced an important economic crisis during these last years, with drastic austerity measures and cuts in several public programs, further decelerating the procedures for refugees.

Refugees and migrants experience extremely stressful events as a result of war, oppression, migration, and resettlement. This includes forced detention, violence, torture, and even witnessing death. UNHCR has repeatedly shared the testimonials of refugees and migrants suffering grave abuses at the hands of smugglers, other criminal networks, and even state authorities [10]. The latter have been implicated in forceful “pushbacks” at land borders, denying refugees access to asylum procedures afforded under European and international law [10]. Travel by sea also poses great risks. The deadliest year for sea crossings was 2016, with 5096 deaths reported [10]. As a result, many refugees and migrants exhibit multiple distressing somatic and psychological symptoms and poor mental health associated with stressful events [11]. A combination of emotional and physical distress is often symptomatic of pre-existing mental health disorders, or a pre-disposition to the development of mental health issues. Studies documenting the types and levels of violence experienced and the prevalence of anxiety and other mental health disorders among Syrian refugees during their displacement and resettlement are lacking [12, 13].

In response to the needs of these displaced populations in Greece and to support the assistance by the Greek population, the international non-governmental organization Médecins Sans Frontières France (MSF) has been supporting refugee camps on mainland Greece, in the regions of Ioannina in northwestern Greece and Attica in the south, as well as a squat-hotel in the center of Athens and a retention center on the Greek island of Samos, providing psychological and medical services. To document the levels and types of violence endured by refugees in their home countries, during their journey, and in their Greek settlements, including the types of perpetrators as well as the prevalence of anxiety and other mental health issues, MSF conducted quantitative and qualitative research at various sites in Greece.

## Methods

### Study settings and participants

We conducted a cross-sectional population-based survey combining quantitative and qualitative components***.*** The parent study took place in eight sites with a larger sample size and recruited multiple nationalities. However, this paper will focus on data from Syrian populations, collected at six different sites representing a range of temporary settlements: (1) the Ritsona camp in the Attica region near Athens, where the population was living in bungalows; (2) the Katsikas camp in Ioannina, in the northwest of Greece; (3) a hotel where a proportion of the Katsikas camp population had been temporarily relocated while the camp was being refurbished for the winter; (4) the island of Samos, one of the first entry points in Greece and a designated UNHCR hotspot; and (5, 6) a pair of squat-hotels in central Athens.

Given the variability of refugee sites in terms of size, structure, and population origin and demographic composition, we opted for an independent sample selection for each study site. For the primary outcome (violence experienced) estimated at 50%, assuming 5% precision, a 5% confidence level and a 10% non-response rate, we estimated a sample size of 250 individuals per site. All enumerated individuals in the randomly or exhaustively (depending on camp size) selected shelters/households were interviewed.

### Study methodology

The quantitative survey included all individuals living in the selected shelter. It consisted of an interviewer-administered questionnaire collecting information on socio-demographic data, journey details, desired country of destination, intended length of stay at the final destination, experience with violence during the journey and in Greece, livelihood, health status, and access to various types of information, mostly legal. The questionnaire was complemented by a pre-validated anxiety disorder screen (the Refugee Health Screener 15 or RHS-15) [14, 15], to detect symptoms of anxiety, depression, and posttraumatic stress disorder, and was administered only to individuals aged 15 years and older. The RHS-15 consists of two (self-administered) components: a set of 13 symptom items scored from 0 to 4 (from “Not at all” to “Extremely”); and a graphic “distress thermometer” scored from 0 to 10. Participants were considered positive when they had a score ≥11 out of 52 in the symptoms component or had a self-reported score ≥5 in the distress thermometer. Any participant who screened positive was informed of the benefit of seeing a mental health professional and provided with a free referral to an on-site psychologist employed by MSF.

The qualitative study included in-depth interviews (IDIs) and focus group discussions (FGDs). The objective of the IDIs was to examine in depth the experience of violence plus mental or psychosocial well-being and coping mechanisms. The objective of the FGDs was to examine access to information and services in relation to asylum procedures. Participants were asked to share reasons for leaving their home country, and the difficulties and violence that they faced during their journey and while in Greece. FGDs and IDIs were audio-recorded and conducted by the qualitative research coordinator with the assistance of translators.

### Data collection and analyses

Quantitative data were collected on paper and entered into the RedCap software. Data quality double-checks were performed on 10% of questionnaires. Descriptive analyses were performed using Stata 13 (Stata Corp). Comparisons of proportions were performed using a chi-squared test. The prevalence of anxiety disorder was stratified by key group characteristics, of which those associated with anxiety were analyzed using a test of proportions.

Audio-recordings of IDIs and FGDs were transcribed into English and reviewed and revised by the qualitative researcher in the field. Qualitative data analyses, aided by NVivo 11 qualitative software, included both thematic analysis and grounded theory [16]. Emergent patterns, categories, and concepts from participants’ accounts were identified by meticulous and systematic reading and coding of the transcripts.

The qualitative data informed the quantitative data and were analyzed simultaneously for the themes chosen for this article.

## Results

### Study participants

**Fig. 1:**
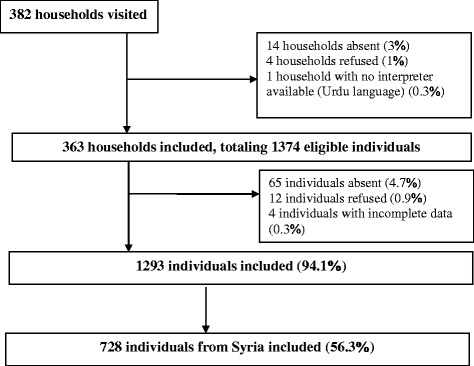
Study flowchart

The study was conducted between 29 November 2016 and 6 February 2017. Of the 382 households eligible and visited, 363 (95%) were included, totaling 1374 eligible individuals, of whom 1293 (94.1%) agreed to participate; only 12 refused (0.9%) (Fig. 1). Of the 1293 consenting participants, 728 (56.3%) were from Syria and analyses were restricted to this population. Similarly, 83 individuals were identified for IDIs/FGDs in the parent study, of whom 42 were of Syrian origin.

The characteristics of the Syrian study population are presented in Table 1. The age and sex composition varied by camp, but overall, they were young (median 18 years, interquartile range 7–30) and predominantly male. The majority of participants aged 15 years and older were married or in unions and a large proportion (42.6–81.8%) had achieved at least secondary education. Most participants were living with their nuclear family at the time of the survey, and 34% originated from Aleppo, 16.8% from Damascus, and 15.6% from Al-Hasakah.

**Table 1:** Population characteristics of Syrian refugees by settlement

Characteristics	Ritsona camp *N* = 286 *n* (%)	Katsikas camp *N* = 133 *n* (%)	Hotel Ioannina *N* = 117 *n* (%)	Samos hotspot *N* = 73 *n* (%)	Athens hotel *N* = 119 *n* (%)	Total *N* = 728 *n* (%)
Female	139 (50.2)	52 (39.7)	53 (45.7)	22 (30.1)	30 (25.2)	296 (41.3)
Median age, years [IQR]	12 [5–30]	21 [9–31]	13 [8–25]	20 [15–31]	23 [17–30]	18 [7–30]
City of origin						
Aleppo	99 (35.5)	39 (29.3)	58 (49.6)	14 (19.2)	35 (29.7)	245 (34.0)
Damascus	26 (9.3)	42 (31.6)	14 (12.0)	15 (20.6)	24 (20.3)	121 (16.8)
Al-Hasakah	61 (21.9)	13 (9.8)	11 (9.4)	18 (24.7)	9 (7.6)	112 (15.6)
Dar Ezor	21 (7.5)	5 (3.8)	11 (9.4)	1 (1.4)	12 (10.2)	50 (6.9)
Family status on site						
Nuclear family	221 (82.2)	92 (71.3)	40 (37.0)	30 (56.6)	27 (25.0)	410 (61.5)
Single parent	9 (3.4)	9 (7.0)	22 (20.4)	1 (1.9)	5 (4.6)	46 (6.9)
Child alone	2 (0.7)	1 (0.8)	–	5 (9.4)	2 (1.9)	10 (1.5)
Alone	10 (3.7)	24 (18.6)	4 (3.7)	16 (30.2)	62 (57.4)	116 (17.4)
Other	27 (10.0)	3 (2.3)	42 (38.9)	1 (1.9)	12 (11.1)	85 (12.7)
*Among respondents ≥ 15 years*						
Marital status						
Single	21 (18.8)	30 (39.5)	21 (39.6)	29 (53.7)	60 (62.8)	161 (41.4)
Married/union	89 (79.5)	45 (59.2)	32 (60.4)	24 (44.4)	34 (36.2)	224 (57.6)
Separated/divorced	1 (0.9)	–	–	–	–	1 (0.3)
Widowed	1 (0.9)	1 (1.3)	–	1 (1.9)	–	3 (0.8)
Unknown	14	2	4	2	1	23
Level of education						
None	15 (13.0)	5 (6.7)	2 (3.6)	3 (5.6)	7 (7.5)	32 (8.2)
Primary	51 (44.4)	11 (14.7)	13 (23.6)	9 (16.7)	10 (10.8)	94 (24.0)
Secondary	32 (27.8)	37 (49.3)	30 (54.6)	33 (61.1)	46 (49.5)	178 (45.4)
Tertiary	17 (14.8)	22 (29.3)	10 (18.2)	9 (16.7)	30 (32.3)	88 (22.5)
Unknown	11	3	2	2	2	20

*IQR* interquartile range

Altogether, 21 IDIs were conducted with Syrians: 11 with a male participant, seven with a female participant, and three with couples. Three FGDs were held, two with women and one with men. In total, 42 Syrians, aged 18–70 years, participated in the qualitative component.

### Experience with violence

The prevalence of participants’ experience with at least one violent event, according to location of occurrence, gender, and age, is presented in Table 2.

**Table 2:** Violence experienced from country of origin to current settlement

Experience of at least one violent event^a^	Ritsona camp *N* = 286 *n* (%)	Katsikas camp *N* = 133 *n* (%)	Hotel Ioannina *N* = 117 *n* (%)	Samos hotspot *N* = 73 *n* (%)	Athens hotel *N* = 119 *n* (%)
In country of origin	85 (30.8)	68 (51.1)	74 (63.3)	55 (77.5)	82 (72.6)
In Turkey	58 (21.3)	36 (28.4)	16 (13.9)	39 (54.2)	23 (19.5)
In Greece	13 (4.6)	9 (6.8)	9 (7.7)	–	8 (6.7)
In site	7 (2.5)	2 (0.3)	10 (8.5)	7 (9.6)	2 (1.7)
During the journey (excluding country of origin)	81 (28.3)	48 (36.1)	29 (24.8)	42 (57.5)	35 (29.4)
Characteristics					
Gender	*p* = 0.82	*p* = 0.73	*p* = 0.43	*p* = 0.39	*p* = 0.59
Men	38/138 (27.5)	28/79 (37.4)	17/63 (27.0)	31/51 (60.8)	25/89 (28.1)
Women	40/139 (28.8)	20/52 (38.5)	11/53 (20.8)	11/22 (50.0)	10/30 (33.3)
Age group	*p* = 0.02	*p* = 0.07	*p* = 0.61	*p* = 0.02	*p* = 0.51
0–5	12 (15.4)	5 (19.2)	3 (16.7)	2 (20.0)	2 (28.6)
6–14	22 (28.6)	9 (31.0)	10 (23.8)	3 (42.9)	3 (17.7)
≥15	42 (33.3)	34 (43.6)	16 (28.1)	37 (66.1)	30 (31.6)
Types of violence experienced in Syria, % of reported violent events					
Bomb	74/112 (66.1)	49/81 (60.5)	48/87 (55.2)	48/80 (60.0)	86/113 (76.1)
Beatings	11/112 (9.8)	15/81 (18.5)	3/87 (3.5)	22/80 (27.5)	13/113 (11.5)
Threats	–	8/81 (9.9)	28/87 (32.2)	2/80 (2.5)	3/113 (2.7)
Traumatized	12/112 (10.7)	5/81 (6.2)	3/87 (3.5)	5/80 (6.3)	1/113 (0.9)
Sexual violence	–	–	–	–	1/113 (0.9)
Other	15/112 (13.4)	4/81 (4.9)	5/87 (5.7)	3/80 (3.8)	9/113 (7.9)
Types of violence experienced in Turkey, % of reported violent events					
Beatings	24/73 (32.9)	4/36 (11.1)	3/16 (18.8)	32/54 (59.3)	2/26 (7.8)
Threats	1/73 (1.4)	14/36 (38.9)	2/16 (12.5)	1/54 (1.9)	5/26 (19.2)
Traumatized	10/73 (13.7)	–	–	5/54 (9.3)	6/26 (23.1)
Sexual violence	–	–	–	–	–
Other	38/73 (52.0)	18/36 (50.0)	11/16 (68.8)	16/54 (29.6)	13/26 (50.0)
Perpetrators of violence experienced in Turkey, % of reported violent events					
Police/army	46/62 (74.2)	22/35 (62.9)	13/17 (76.5)	47/53 (88.7)	16/27 (59.3)
Smugglers	16/62 (25.8)	11/35 (31.4)	4/17 (23.5)	2/53 (3.8)	4/27 (14.8)
Other refugees	–	–	–	1/53 (1.9)	–
Local population	–	1/35 (2.9)	–	2/53 (3.8)	7/27 (25.9)
Others	–	1/35 (2.9)	–	1/53 (1.9)	–
Types of violence experienced in Greece, % of reported violent events					
Beatings	14/17 (82.3)	8/10 (80.0)	8/10 (80.0)	3/7 (42.9)	4/12 (33.3)
Threats	–	–	–	2/7 (28.6)	4/12 (33.3)
Tear gas	–	1/10 (10.0)	2/10 (20.0)	–	–
Sexual violence	–	–	–	–	–
Other	3/17 (17.7)	1/10 (10.0)	–	2/7 (28.6)	4/12 (33.3)
Perpetrators of violence experienced in Greece, % of reported violent events					
Police/army	6/17 (35.3)	5/7 (71.4)	2/10 (20.0)	2/8 (25.0)	8/12 (66.7)
Smugglers	–	–	–	–	–
Other refugees	7/17 (41.2)	1/7 (14.3)	8/10 (80.0)	2/8 (25.0)	3/12 (25.0)
Local population	–	–	–	4/8 (50.0)	1/12 (8.3)
Others	3/17 (17.7)	1/7 (14.3)	–	–	–

^a^A violent event relates to any violence experienced such as kidnapping, tear gas, bomb, physical torture, trauma (mental shock), knife or other weapon use, sexual violence, beating, or other

The majority of participants experienced at least one violent event in Syria, ranging from 30.8% of those at Ritsona camp to 77.5% at the Samos hotspot. During the journey (excluding in Syria), between 24.8% (Hotel Ioannina) and 57.5% (Samos) of interviewees experienced at least one violent event. Approximately one-quarter of participants at Ritsona and Katsikas camps and the Athens hotel, and more than half of participants at the Samos hotspot, experienced a violent event in Turkey; and between 4.6% (Ritsona) and 7.7% (Hotel Ioannina) in Greece; while the proportion experiencing violence at their current settlement ranged from 0.3% (Katsikas) to 9.6% (Samos). Among men and women, similar proportions of violence experienced were observed but across all sites, a greater proportion of the older age group reported a violent event. Violent events reported in Syria were mainly bombing of the cities (55.2–76.1%) and threats (2.5–32.2%). The types of violence in Turkey and Greece were mainly beatings (7.8–59.3% in Turkey and 33.3–82.3% in Greece), perpetrated by police in both countries but also by other refugees in Greece. Furthermore, psychological trauma from witnessing severely distressing events constituted up to 13.3% of the violent events reported in Turkey.

Many of the participants interviewed had experienced different forms of violence, for example torture and bombing of their houses. Some had been detained, and others not only lost property but also family members and no longer felt they had any reason to remain at home. The perpetrators of the violence in Syria were mainly the regime or Daesh:

*[Daesh] brought me to in a room, where I had to turn against the wall and raise my arms. Then they started whipping me. I was pregnant, eight months pregnant.* (Woman from Syria)

Participants highlighted difficulties and violence when crossing checkpoints inside Syria. In addition, when trying to enter Turkey, participants were often shot at by the Turkish police and border guards:

*[The police] fired at us and some people were injured or killed. Others crossed the border and others were caught by Turkish border guards and pushed back to Syria again.* (Palestinian man from Syria)

During the journey, participants interviewed in the qualitative study often found themselves in situations over which they could exercise little or no control and where they received limited information from smugglers. The ability to negotiate with smugglers was complicated by the practice of smugglers handing over people to other smugglers, through chains of delegation. In addition, families were at times separated. One participant described how he and the rest of his family were separated from their 2-year-old son while crossing the border to Turkey:

*One guy was carrying one of my sons when we walked through the mountains as I couldn’t carry both. […] We got lost because of the huge numbers of people who were trying to cross the borders. The Turkish police caught this guy with my son and sent them back to Syria.* (Man from Syria)

Similarly, participants were threatened with guns by the smugglers and shot at by the Turkish Coast Guard when crossing by sea from Turkey to Greece. When crossing into Greece by land, participants also faced violence if caught. Participants mentioned tensions and violent episodes in the camp, making them worry about the safety of themselves and their families. Living in tents and the strong feeling of not being protected by the police increased the feeling of insecurity.

### Mental health and referral and acceptance of psychological assessment

The results of the RHS-15 screening tool for anxiety disorder morbidity are presented in Table 3, according to participant characteristics and site, as well as the levels of acceptance of referral for mental health evaluation. For each individual characteristic, the percentages of participants screened positive are presented. The tool was administered to 80.5% (332/412) of the respondents aged 15 years and over.

**Table 3:** Prevalence of anxiety disorder morbidity

Refugee Health Screener 15	Ritsona camp *N* = 100 *n* (%)	Katsikas camp *N* = 67 *n* (%)	Hotel Ioannina *N* = 48 *n* (%)	Samos hotspot *N* = 38 *n* (%)	Athens hotel *N* = 79 *n* (%)
Screened positive	80 (80.00)	50 (74.6)	36 (75.0)	35 (92.1)	60 (76.0)
Women	40 (83.3)	19 (82.6)	23 (82.1)	10 (100.0)	11 (91.7)
Men	39 (76.5)	30 (69.8)	13 (65.0)	25 (89.3)	49 (73.1)
Age group					
14–25	21 (77.8)	20 (76.9)	16 (69.6)	19 (95.0)	31 (75.6)
>25	59 (80.8)	30 (73.2)	20 (80.0)	16 (88.9)	29 (76.3)
Marital status					
Single	14 (70.0)	22 (73.3)	12 (66.7)	19 (90.5)	39 (76.5)
Married/union	59 (80.8)	26 (74.3)	21 (77.8)	15 (93.8)	20 (74.1)
Separated/divorced	1 (100.0)	1 (100.0)	–	1 (100.0)	–
Widowed					
Family status at the site					
Nuclear family	56 (80.0)	25 (71.4) to	12 (63.2)	7 (100.0)	8 (80.0)
Single parent	7 (100.0)	6 (100.0)	13 (86.7)	1 (100.0)	4 (80.0)
Child alone	2 (100.0)	1 (100.0)	–	4 (100.0)	1 (100.0)
Alone	7 (77.8)	16 (72.7)	1 (50.0)	11 (91.7)	42 (76.4)
Family in Europe					
Yes	70 (79.6)	44 (75.9)	32 (76.2)	29 (90.6)	55 (78.6)
No	10 (83.3)	6 (66.7)	3 (60.0)	6 (100.0)	5 (55.6)
Experienced at least one violent event					
Yes	25 (83.3)	21 (72.4)	9 (75.0)	20 (90.9)	18 (75.0)
No	55 (78.6)	29 (76.3)	27 (75.0)	15 (93.8)	42 (76.4)
Chronic disease^a^					
Yes	8 (100.0)	3 (100.0)	6 (100.0)	4 (100.0)	7 (70.0)
No	72 (78.3)	47 (73.4)	30 (71.4)	31 (91.2)	53 (76.8)
Vulnerable^b^					
Yes	16 (94.2)	8 (100.0)	13 (76.5)	5 (100.0)	5 (71.4)
No	64 (77.1)	42 (72.2)	23 (74.2)	30 (90.9)	55 (76.4)
Length of stay in Greece					
>9 months	22 (88.0)	18 (72.0)	30 (75.0)	2 (100.0)	55 (76.4)
≤9 months	56 (76.7)	32 (76.2)	6 (75.0)	33 (91.7)	5 (71.4)
Duration of travel					
>2 months	46 (85.2)	24 (75.0)	18 (94.7)	13 (92.9)	30 (69.8)
≤2 months	30 (73.2)	26 (74.3)	18 (62.1)	22 (91.7)	29 (82.9)
Women vs Men	*p* = 0.40	*p* = 0.26	*p* = 0.18	*p* = 0.28	*p* = 0.17
14–25 years vs >25 years	*p* = 0.74	*p* = 0.73	*p* = 0.40	*p* = 0.49	*p* = 0.94
Young women (14–25) vs older women	*p* = 0.47	*p* = 0.77	*p* = 0.83	–	*p* = 0.46
Young men (14–25) vs older men	*p* = 0.83	*p* = 0.71	*p* = 0.66	*p* = 0.46	*p* = 0.97
Having family in Europe vs no family in Europe	*p* = 0.76	*p* = 0.56	*p* = 0.43	*p* = 0.43	*p* = 0.13
Experienced at least one violent event vs no violent event	*p* = 0.59	*p* = 0.72	*p* = 0.99	*p* = 0.75	*p* = 0.90
Chronic disease vs no chronic disease	*p* = 0.14	*p* = 0.30	*p* = 0.13	*p* = 0.54	*p* = 0.64
Vulnerable vs not vulnerable	*p* = 0.11	*p* = 0.08	*p* = 0.86	*p* = 0.48	*p* = 0.77
Stayed in Greece <9 vs >9 months	*p* = 0.23	*p* = 0.70	*p* = 0.99	*p* = 0.67	*p* = 0.77
Traveled >2 vs <2 months	*p* = 0.15	*p* = 0.95	*p* = 0.01	*p* = 0.90	*p* = 0.18
Among those screened positive:					
Declined referral	19/80 (23.8)	12/50 (24.0)	9/48 (25.0)	11/35 (31.4)	11/60 (18.3)

^a^A chronic disease is any disease requiring chronic treatment

^b^Vulnerable populations include pregnant women, children alone, single parents, and those with a self-reported chronic disease or ≥60 years old

The vast majority of participants screened positive for anxiety disorder meriting referral for a mental health evaluation: nearly all participants (92.1%) living on Samos, 80.0% of the respondents from the Ritsona camp, and approximately three-quarters of respondents from Katsikas camp, Hotel Ioannina and Athens hotel (74.6%, 75.0%, and 76.0%, respectively). The prevalence of anxiety disorders was high, similar by sex and varied slightly by age group. No differences between individual characteristics and prevalence of anxiety were observed, except in Hotel Ioannina where refugees who had traveled for more than 2 months had a higher prevalence of anxiety compared to refugees who had traveled for less than 2 months (*p* = 0.01). About one-quarter of participants in Ritsona and Katsikas camps and Hotel Ioannina declined referral. The proportion declining referral was higher (31.4%) on Samos and lower (18.3%) at the Athens hotel.

While war, violence, and harsh conditions during the journey stood out as traumatic experiences for most, the qualitative study participants emphasized that their current lives as refugees in Greece and the uncertainty about their futures were especially detrimental to their mental well-being. The refugees in Greece described their current lives as a source of pain and suffering. Their new lives and identities as refugees were very different and poor compared to their previous lives in Syria before the civil war. Living or having lived in poor housing conditions for several months (tents, containers, etc.) in often isolated camps with movement restrictions, and denial of or inability to exercise the same rights as the surrounding Greek society, made refugees feel socially marginalized and discriminated against. Some described how they had lost their dignity, felt humiliated, or felt treated worse than animals:

*I was really humiliated here [in Greece], and I have even experienced the bombing in Syria. However, I still had dignity there [in Syria]. Here, I lost it completely. When you have to stay [a] long time in line just to bring food to your children. My son asks me for some chips, but I can’t buy them for him. In Syria, I was buying everything: clothes, food, everything.* (Man from Syria)

Being separated from family members was another issue that caused emotional suffering among Syrian refugees in Greece. Some had been separated during the journey, while others were separated when certain family members did not travel, remaining behind in their home country. Several qualitative study participants experienced being separated within Greece from their adult children, adult siblings, or other family members not defined as core family (spouses and under-aged children). While the new life situation as a refugee in Greece was already difficult, not being with family members or other social support networks further worsened the situation.

### Legal procedures and access to information in Greece

Table 4 presents the initiation of legal procedures and access to information on legal assistance, asylum procedures, and healthcare. On Samos, the majority of participants (86.1%) reported having initiated the asylum procedure. At the Ritsona and Katsikas camps and the Athens hotel, most participants had started the relocation procedure (70.0%, 73.2% and 71.8%, respectively). The highest proportion of participants (60.7%) seeking reunification were at Hotel Ioannina. However, 3.7% of participants at Ritsona, 4.2% at the Samos hotspot, and 7.1% at Katsikas camp did not know which procedure to pursue. At the time of the study, the majority of respondents were waiting for an answer regarding the outcome of their legal procedure (between 77.8% at the Athens hotel and 100% at both the Katsikas camp and Hotel Ioannina).

**Table 4:** Legal procedures initiated and access to legal information

	Ritsona camp *N* = 286 *n* (%)	Katsikas camp *N* = 133 *n* (%)	Hotel Ioannina *N* = 117 *n* (%)	Samos hotspot *N* = 73 *n* (%)	Athens hotel *N* = 119 *n* (%)
Procedure initiated					
Asylum in Greece	19 (7.0)	2 (1.6)	–	62 (86.1)	12 (10.3)
Relocation	189 (70.0)	93 (73.2)	46 (39.3)	–	84 (71.8)
Reunification	52 (19.3)	21 (16.5)	71 (60.7)	6 (8.3)	21 (18.0)
None	–	2 (1.6)	–	1 (1.4)	–
Don’t know	10 (3.7)	9 (7.1)	–	3 (4.2)	–
Status of procedure					
Don’t know/in progress	127 (95.5)	99 (100.0)	77 (100.0)	52 (96.3)	84 (77.8)
Accepted	6 (4.5)	–	–	1 (1.9)	21 (19.4)
Rejected	–	–	–	1 (1.9)	3 (2.8)
Considered had received necessary information about:					
Legal assistance	44 (15.4)	40 (30.1)	2 7(23.1)	7 (9.6)	17 (14.3)
Procedure for asylum	47 (16.4)	42 (31.6)	44 (37.6)	8 (11.0)	17 (14.3)
Access to healthcare	222 (77.6)	101 (75.9)	89 (76.1)	44 (60.3)	101 (84.9)

A very low proportion of participants reported having had access to information on legal assistance, between 9.6% (Samos) and 30.1% (Katsikas.) Information on asylum procedures was also generally limited, with only 11.0% (Samos) to 31.6% (Katsikas) of the population considering that they had received the necessary information. Conversely, access to information on where to obtain healthcare was high, ranging from 60.3% (Samos) to 84.9% (Athens hotel).

For participants, at the time of the study in Greece, knowing what would happen to them and their families mattered the most and they often directly linked uncertainty to poor psychological well-being:

*Will they accept you in Europe? And when will they accept you? Only God knows. I am getting mentally ill because I have been in this situation for 10 months.* (Man from Syria)

*When is this game ending? When are we going [to get] out of this place and out from Greece? Most of us have someone in Europe, a child or a husband, otherwise we could go back to our country: We prefer to die 60 times and not to be stuck here.* (Woman from Syria)

Participants interviewed in the qualitative study said that the lack of guidance and information on asylum procedures increased their feelings of uncertainty about the future, which was taking a toll on their mental and psychosocial well-being.

When seeking information about asylum options and consequences, participants did not receive the guidance and information they sought. They mentioned leaving the services offered by UNHCR or European Asylum Support Office in the camps without getting their questions answered. Consequently, participants asked for advice from peer refugees and migrants in similar situations. All of the Syrians wanted guidance on procedures for family reunification or relocation to another EU country:

*We did the interview for the reunification program. However, my daughter was over 18 years and not eligible, which they did not inform us about at that time. We were waiting and after 4 months, they told us this, and asked us to apply for the relocation program. Now all our procedures are stuck and when we go to the asylum office to ask, they do not allow us to enter and they give us no information at all.* (Woman from Syria)

When describing their current situation in Greece, participants often employed such terms as “hopelessness” and “losing hope.” On Samos and among the unregistered migrants in Greece, respondents described their situation as extremely stressful:

*The fear [of being deported] is always there, and always I find myself stressed. I try to forget, not to think about the issues. I am on my nerves all the day, thinking is the lawyer going to call me, I never leave my phone.* (Woman from Syria)

## Discussion

To our knowledge, this is the first study conducted in Greece using a mixed quantitative and qualitative methodology to describe experiences with violence and the prevalence of anxiety disorders amongst Syrian (and other) refugees and migrants. Our findings provide evidence of the high levels of distress caused by war, oppression, migration, resettlement, and uncertainty about the future experienced by Syrian refugees and migrants. The presence of many families in the study sites, not only of single young men, explains the low average age of the study population and corroborates the main reasons provided by participants for migration (i.e., their vital need to flee a horrifying conflict in their home country with high levels of violence, rather than to seek economic gain). Indeed and importantly, our findings document the multiple types and levels of violence endured by these populations when away from home, during their journeys, and even once settled in Turkey and Greece. The low levels of access of refugees and migrants to information, coupled with the extremely long bureaucratic procedures for seeking and obtaining asylum, compound the hardships endured by this population. Humanitarian and political assistance is urgently needed to curb the violence and provide structured protective, medical, and psychological support.

One of the key objectives of the study was to document the prevalence and types of violence experienced by the refugees in their country of origin, during the journey, and also, tragically, in Greece. Our description of the reported violence is highly detailed in terms of time and place, but also in terms of type and perpetrator. For each site, the rate of violence experienced decreased during the journey, starting at a high level in Syria, where conflict was significant, to a lower level in Greece, a European country with no conflict, but where nevertheless, violent events were reported. Refugees fleeing war and threats [17] faced violence in their country of origin and continued to experience violence during their journeys and in Greece. In Turkey and in Greece, the majority of the types of violence reported were beatings, perpetrated by the police but also by other refugees in Greece. Specific examples were reported during the qualitative component of our study. A report published in 2016 by the organization Human Rights Watch has specifically documented and condemned abuse from the Turkish police and coast guard against Syrian refugees [18].

One of the main objectives of the study was to document the mental health of the refugee population in the settlement sites, using a screening tool that detects the symptoms of anxiety and depression among refugees. The study highlights a high prevalence of positive screening using this anxiety disorder tool. While this may reflect the truly astonishingly high levels of distressing events encountered by this population, it may also be that the tool is too sensitive and not specific enough for a refugee population living in difficult material conditions with high levels of recent trauma [19]. Indeed, we did not observe significant differences between individual characteristics and level of anxiety, suggesting that the level of anxiety and depression observed may be attributed to living conditions and uncertainty about the future. Numerous other studies confirm that daily stressors, defined as everything from poverty, social marginalization, isolation, and inadequate housing to changes in family structure and functioning, contribute to the high rate of psychological distress often found in conflict-driven migrants [20–22]. The purpose of the screening tool is not to provide a diagnosis, but rather to offer a referral opportunity to a mental health specialist such as our on-site psychologists. The rate of declining a referral was high in all sites. This could be explained by cultural perceptions of psychological services or by a lack of trust among refugees struggling with legal procedures who are unwilling to discuss issues with a psychologist without receiving procedural advances. Furthermore, in the context of camps with communities living in close proximity, rumors and the fear of stigma and discrimination may act as disincentives for open consultations with psychologists.

Analyses of the legal aspects show a lack of information and feedback being given to refugees. At all sites, the majority of respondents did not know the status of their applications, with many having pending procedures at the time of the survey (late 2016). This protracted process and lack of communication can only exacerbate the distress experienced by refugees. The finding that information about legal assistance and procedures for asylum was non-existent exposes the shameful state of an overloaded and disorganized administration in Greece, a country that has faced drastic austerity measures, resulting in cuts to public employees. In particular, our study corresponds with another MSF report that showed there was an inefficient relocation system [23]. Furthermore, research has demonstrated that lack of legal assistance and long asylum procedures are important risk factors for anxiety and psychological distress among asylum seekers in high-income countries [24, 25].

The parent study of this report was conducted among all refugees and migrants residing in the same study sites. The findings for the Syrian population do not differ substantially from those observed among the whole study population [26], highlighting the importance of the circumstances of living in camps in addition to the traumatic journey experience. Similar findings were reported in a study conducted by MSF Belgium in Serbia between 2015 and 2016, which documented a high incidence of violent events experienced by refugees traveling through the Balkans to Northern Europe [27].

An important study limitation not already mentioned was the difficulty of documenting individual histories, including the complexity of reporting sexual violence. Barriers to reporting sexual violence among Syrian populations have been documented [28]. Importantly, as our study population traveled in groups, often as family units, they may have been less vulnerable and at risk of sexual assault. It is also possible that sexual assault might not be that common on the routes we described, compared to refugees and migrants traveling through other areas, such as Libya [29].

## Conclusions

In conclusion, this report highlights extremely high levels of violence experienced by Syrian refugees during their journeys and when seeking protection in Greece, including violence perpetrated by some state authorities. Unsurprisingly, the report also highlights high levels of anxiety and distress in this population, compounded by lack of information on legal procedures and uncertainty about the future. A comprehensive humanitarian and political response is urgently needed to provide and protect basic humanitarian rights and refugee laws and provide the care and compassion owed to traumatized populations.

## Abbreviations

EU: European Union
FGD: Focus group discussion
IDI: In-depth interview
MSF: Médecins Sans Frontières
RHS: Refugee Health Screener
UNHCR: United Nations High Commissioner for Refugees
